# Perpendicular magnetic tunnel junction with a strained Mn-based nanolayer

**DOI:** 10.1038/srep30249

**Published:** 2016-07-26

**Authors:** K. Z. Suzuki, R. Ranjbar, J. Okabayashi, Y. Miura, A. Sugihara, H. Tsuchiura, S. Mizukami

**Affiliations:** 1 WPI Advanced Institute for Materials Research, Tohoku University, Sendai 980-8577, Japan; 2 Research Center for Spectrochemistry, University of Tokyo, Tokyo 113-0033, Japan; 3 Electrical Engineering and Electronics, Kyoto Institute of Technology, Kyoto 606-8585, Japan; 4 Department of Applied Physics, Tohoku University, Sendai 980-8579, Japan

## Abstract

A magnetic tunnel junction with a perpendicular magnetic easy-axis (p-MTJ) is a key device for spintronic non-volatile magnetoresistive random access memory (MRAM). Co-Fe-B alloy-based p-MTJs are being developed, although they have a large magnetisation and medium perpendicular magnetic anisotropy (PMA), which make it difficult to apply them to a future dense MRAM. Here, we demonstrate a p-MTJ with an epitaxially strained MnGa nanolayer grown on a unique CoGa buffer material, which exhibits a large PMA of more than 5 Merg/cm^3^ and magnetisation below 500 emu/cm^3^; these properties are sufficient for application to advanced MRAM. Although the experimental tunnel magnetoresistance (TMR) ratio is still low, first principles calculations confirm that the strain-induced crystal lattice distortion modifies the band dispersion along the tetragonal *c*-axis into the fully spin-polarised state; thus, a huge TMR effect can be generated in this p-MTJ.

Magnetic tunnel junctions (MTJs) composed of two magnetic layers separated by a thin insulating barrier, such as Al-O or MgO, exhibit tunnel magnetoresistance (TMR), depending on the relative orientation of magnetisation^1^,^2^,^3^,^4^. MTJs with a magnetic layer with a perpendicular easy-axis of magnetisation (p-MTJs) become key devices for the realisation of high recording density non-volatile memory by using the spin-transfer-torque (STT) effect^5^,^6^. The magnetisation direction can be efficiently controlled in such p-MTJs by applying the electric current; thus they can be used to realise STT-magnetoresistive random access memory (STT-MRAM). STT-MRAM has unique properties, i.e., non-volatility, scalability, high speed, and low consumption power, that have never been obtained in non-magnetic devices or systems^7^,^8^. Currently, Co-Fe-B alloy and its derivatives are widely used for p-MTJs because they possess high spin-polarisation, which leads to a large TMR effect^9^,^10^. However, the alloy’s large saturation magnetisation of approximately 1000 emu/cm^3^, which results from the main constituents of Fe-Co, makes the p-MTJs difficult to integrate with higher density and faster writing speed^8^,^11^. In addition, its perpendicular magnetic anisotropy (PMA), which originates from the MgO/Co-Fe-B interface, with a typical value of 1–2 Merg/cm^3^, is not large enough to retain the magnetisation direction against thermal fluctuation when the p-MTJ size is reduced to 10–20 nm^8^. Thus, the exploration of special magnetic materials for p-MTJs to overcome such limitations is required.

The ordered tetragonal Heusler-like Mn-based alloys, such as Mn_3_Ga and its derivatives, have attracted much attention for STT-applications because they have high spin-polarisation related to the Heusler structure and low saturation magnetisation due to ferrimagnetism^12^,^13^,^14^,^15^,^16^,^17^,^18^. A high bulk PMA and low Gilbert damping constant also originate from the special property of Mn, i.e., it has nearly half-filled 3*d* electron orbital states in a crystal field with tetragonal symmetry^19^. In addition, those tetragonal Mn-based alloy films also exhibit high PMA fields of 60–200 kOe owing to the low magnetisation, which enables long-lifetime magnetisation precession at a terahertz (THz) frequency^20^,^21^, and thus they can be applied to STT-oscillators and diodes in the THz frequency range^22^,^23^. One technological challenge is to realise p-MTJs with an ultrathin Mn-based alloy layer with a large PMA and a typical thickness of 1–3 nm. This is crucial for devices driven by the STT effect. However, this has not yet been achieved, because growth of Mn-based alloy nanolayers on conventional buffer layers, such as Cr, has deteriorated their PMA^24^,^25^,^26^,^27^,^28^.

Here we successfully demonstrate p-MTJs with 3-nm-thick MnGa layers with L1_0_ chemical ordering. This was achieved by means of an RT growth process of MnGa in combination with a unique material, CoGa, that is paramagnetic at RT^29^, in a buffer layer, which enabled us to obtain an atomically flat interface of the MnGa layer with an epitaxial strain. The MnGa layer exhibits high PMA and low saturation magnetisation, even in strained states. Even though the experimental TMR ratio is still low, the first principles calculations confirm that the strained MnGa has fully spin-polarised band dispersion along the tetragonal *c*-axis; this is distinct from bulk Mn-Ga and similar to Co-Fe(-B)^3^,^4^,^30^,^31^ and Mn_3_Ge^32^,^33^,^34^, which implies that a huge TMR is possible.

## Results

### MTJ stacking and crystal structures

**Figure 1:**
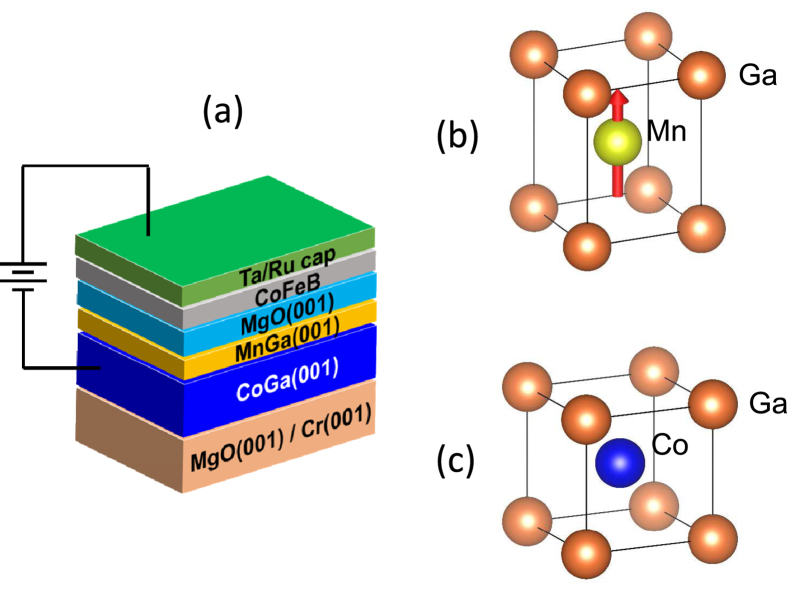
Perpendicular magnetic tunnel junction (p-MTJ) stacking and crystal structures. Schematic of p-MTJ multilayer stacking structure (**a**) and crystal structures of the L1_0_ ordered tetragonal MnGa (**b**) and the paramagnetic B2 ordered cubic CoGa (**c**). The arrow denotes the magnetic moment of the Mn atom. The L1_0_ crystal unit cell is considered as a body-centred tetragonal (bct) cell to easily compare it with the bcc unit cell of CoGa.

The p-MTJ stacking structure of Cr(40)/Co_55_Ga_45_(30)/Mn_60.5_Ga_39.5_(3)/MgO(2)/Co_20_Fe_60_B_20_(1)/Ta(3)/Ru(5) (thickness in nm) was prepared on a MgO (100) single crystalline substrate, as schematically shown in Fig. 1. Here, CoFeB is a top magnetic electrode, and Ta and Ru are the capping layers. The p-MTJ was not annealed after microfabrication to avoid atomic diffusion. The crystal structures of bulk CoGa and MnGa are B2 and L1_0_, respectively, as also shown in Fig. 1. The L1_0_ structure can be regarded as a tetragonal B2 structure. Although there is a lattice mismatch between cubic CoGa and tetragonal MnGa, MnGa can be epitaxially grown on CoGa in cube-on-cube with epitaxial strain, as mentioned below.

### Structural characterisation

**Figure 2:**
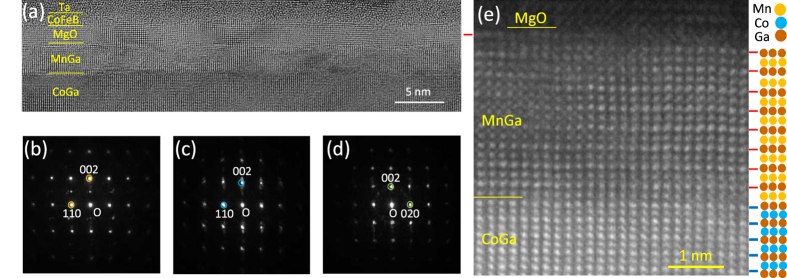
Nano and atomic structures of the MTJ multilayer stacking by TEM. (**a**) Cross-sectional HRTEM image of the CoGa/MnGa/MgO/CoFeB/Ta region of MTJ stacking structure. The nano-beam diffraction patterns of (**b**) CoGa, (**c**) MnGa, and (**d**) MgO layer, where the diffraction planes are indicated in the photographs. (**e**) The HAADF-STEM image of the CoGa/MnGa/MgO region and the corresponding atom of Co, Ga, and Mn are schematically indicated be the coloured solid circles.

Figure 2(a) shows a cross-sectional image of an MTJ stack taken by high-resolution transmission electron microscopy (HRTEM). A (001)-oriented epitaxial growth from the CoGa buffer to the MgO barrier layer is clearly observed. In particular, the interface between the CoGa and MnGa layers is well-defined due to an atomically smooth surface of the CoGa layer, and no significant defects at the interface are seen. The lattice parameters were evaluated from the nano-beam electron diffraction patterns for each layer, as shown in Fig. 2(b–d), and these are summarised in Table 1 with the bulk values^35^,^36^,^37^. The lattice parameters for the CoGa layer and MgO barrier were nearly identical to the bulk values. The in-plane lattice parameter of the MnGa layer is close to that of the CoGa layer and the unit-cell volume evaluated for the MnGa layer is nearly equal to that of bulk. This indicates that the MnGa layer is grown with a reduction of its tetragonal axial ratio so as to fit to the lattice of the CoGa buffer layer.

The structure of the CoGa/MnGa/MgO region is clarified in more detail by atomic imaging by high-angle annular dark-field scanning transmission electron microscopy (HAADF-STEM), as shown in Fig. 2(e), where the periodic arrangement of atoms of the CoGa and MnGa layers are clearly visible. The bright spots are identified as Ga atoms because the atomic number of Ga is significantly different from that of Co and Mn. On the other hand, the relatively dark spots in each layer correspond to Co or Mn, whose element selectivity indicates relatively homogeneous epitaxial strain as well as no significant site swapping in each unit cell nor diffusion at the CoGa/MnGa interface. In the CoGa layer, the bright and dark spots alternately align to the (001) direction, which results from the well-ordered B2 structure. It can be seen that the interface of the CoGa layer is terminated by a Ga-rich atomic plane, on which the MnGa layer is formed. The dark and bright spots also alternately align in the MnGa layer, similarly to the CoGa layer, clearly showing the chemical ordering of MnGa in spite of no heat treatment in preparation process of the MnGa. It should be noted that the RT-growth of the MnGa layer with better chemical ordering was obtained on the CoGa buffer layer annealed only at high temperature^29^. This implies that the growth of MnGa with a layer-by-layer structure of Mn and Ga atomic planes may be promoted by the Ga atomic plane terminated-interface of the CoGa layer, and thus it is speculated that the termination element is important for the growth mode of the MnGa layer. On the other hand, the bright spots are remarkably seen at MnGa/MgO interface, which suggests that this interface is terminated by the Ga atomic plane.

### Characterization by X-ray spectroscopies

**Figure 3:**
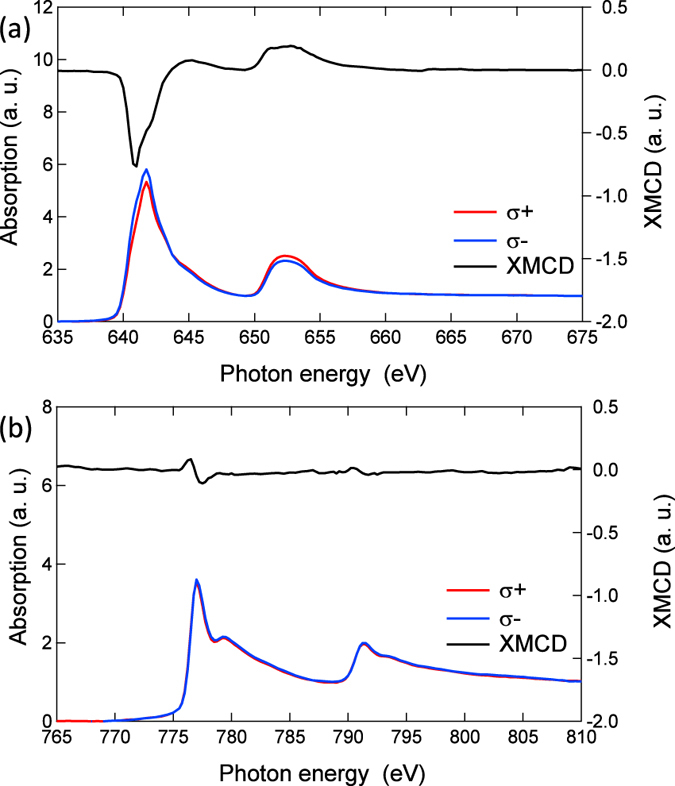
High energy X-ray characterisation of CoGa/MnGa/MgO layers of the p-MTJ. The X-ray absorption spectra (XAS) and X-ray magnetic circular dichroism for (**a**) Mn and (**b**) Co *L*_2,3_ edges for the blanket film of the MTJ stacking without the top CoFeB and capping layers.

The valence states and magnetic state of 3*d* electron orbits for Mn and Co were investigated using X-ray absorption spectroscopy (XAS). The spectral lines for the Mn and Co *L*_2,3_-edges for the MTJ stacking films without the top CoFeB/Ta/Ru layer are shown in Fig. 3(a,b), respectively. The X-ray penetration depth in this measurement is approximately 5 nm, so that the measurement probes the entire MnGa layer and the interface region of the CoGa buffer layer within a depth of several nm. The broad line shape for the Mn *L*-edge and its X-ray magnetic circular dichroism (XMCD) are similar to those reported for thick Mn-Ga films^38^. The Co *L*-edge exhibits the same shape as that of the CoGa control film and negligible variations with different polarisations of the incident X-ray beam. This absence of XMCD for Co is further evidence for a lack of diffusion of Co into MnGa, because Co has a magnetic moment and shows XMCD when it is doped in Mn-Ga alloys^39^.

### Magnetic and transport properties

**Figure 4:**
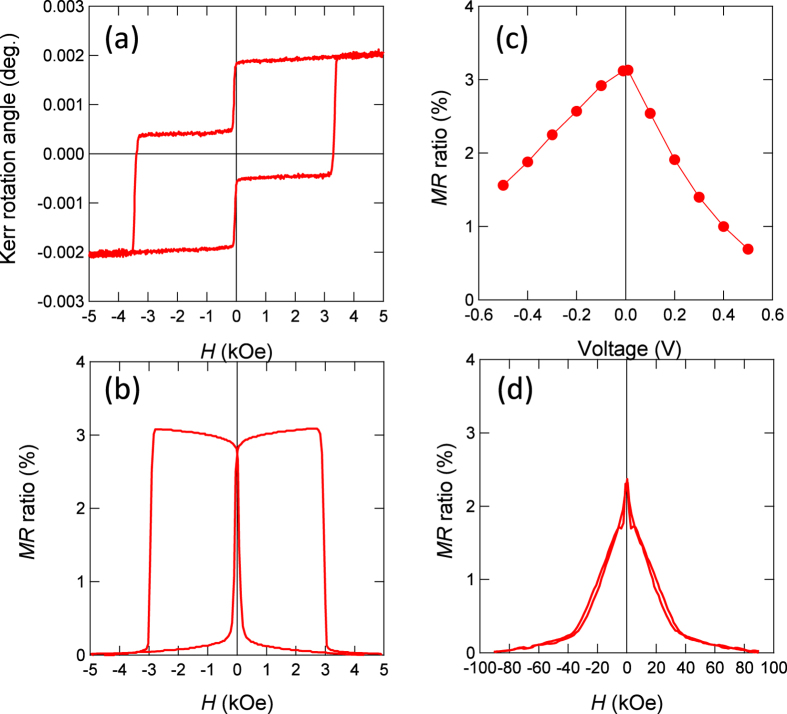
Magnetic and magnetoresistive properties at RT. (**a**) The out-of-plane MOKE hysteresis curve of the MTJ stacking structure. (**b**) The TMR curve measured while applying magnetic field perpendicular to the film plane and (**c**) its bias voltage dependence of the TMR ratio measured at 300 K. (**d**) In-pane TMR curve.

Figure 4(a) shows the polar magneto-optical Kerr effect (MOKE) hysteresis loop for the blanket film for MTJs. Abrupt magnetisation switching between the parallel and anti-parallel states was observed and is in accord with the small and large coercive forces for the CoFeB and MnGa layers, respectively. A TMR curve measured at 300 K with magnetic field applied perpendicular to the film plane is shown in Fig. 4(b), which corresponds to the magnetisation process shown in Fig. 4(a). The bias voltage dependence of the TMR ratio measured at 300 K is plotted in Fig. 4(c). Here, the sign of the bias voltage is defined as positive with respect to the CoFeB layer (Fig. 1). An asymmetry with respect to the bias polarity is observed, similar to that of the Fe/MgO/Fe MTJs^3^. For negative bias voltage, the TMR ratio decreases more gradually with increasing bias than for positive bias voltage. The TMR ratio at the bias voltage of approximately +0.3 V and −0.5 V becomes half of the maximum value. Although the TMR ratio for this p-MTJ of approximately 3% is small, it is comparable to that of previously reported similar p-MTJs with much thicker MnGa electrodes^40^.

A TMR curve measured while applying magnetic field in the film plane is shown in Fig. 4(d). It shows a maximum at near zero field, a gradual decrease with increasing magnetic field, and a kink at approximately 40 kOe. This gradual change and kink correspond to the magnetisation rotation and saturation of the MnGa layer, respectively, since the PMA field of the CoFeB layer is several kilooersteds. The effective PMA field value 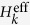 for the MnGa layer was estimated from the value corresponding to the field at the kink, and the large value of 36 kOe is found. The saturation magnetisation *M*_*s*_ for the reference sample, which was a 3-nm-thick MnGa layer grown on a CoGa buffer and capped by a MgO layer was evaluated to be approximately 350 ± 50 emu/cm^3^; the effective PMA constant 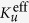 for the 3-nm-thick MnGa layer in the p-MTJ was estimated to be 6.3 ± 0.9 × 10^6^ emu/cm^3^ using the relation 
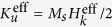
. This 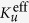 value is much greater than that for the 3-nm-thick MnGa nanolayer grown on a Cr buffer layer reported previously^26^. It is also larger than the typical values of Ta/Co-Fe-B/MgO^10^ and is comparable to those for Co/Pt and Co/Pd multilayer films even though the saturation magnetisation is much smaller than for those material films^41^.

**Figure 5:**
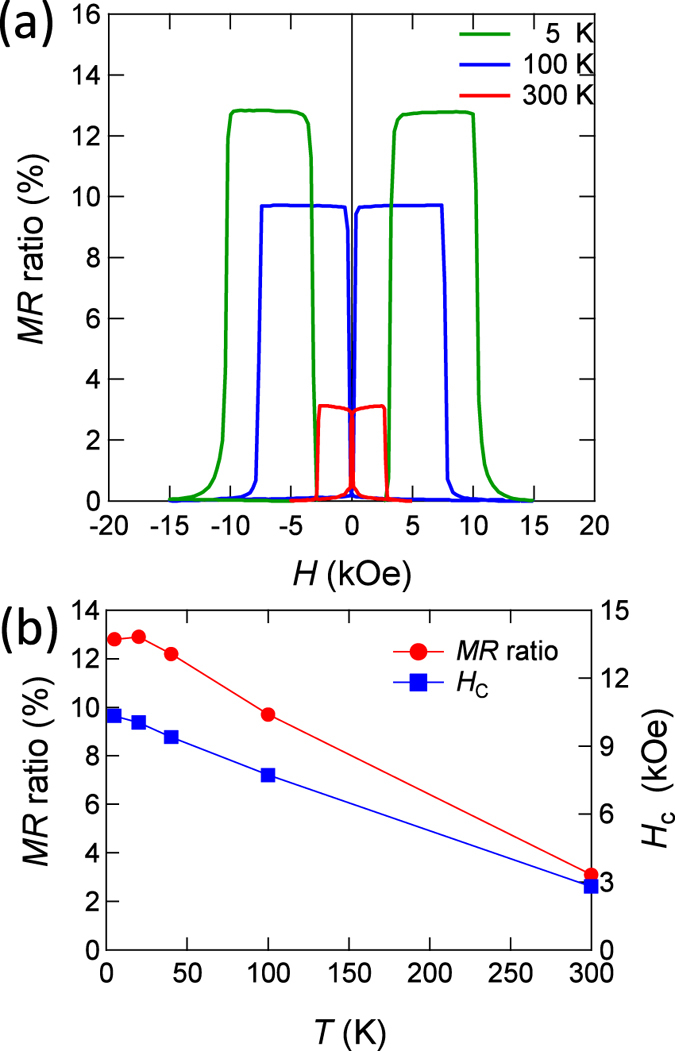
Temperature dependence of the TMR effect. (**a**) TMR curves measured at temperatures of 5, 100, and 300 K while applying magnetic field perpendicular to the film plane. (**b**) Temperature dependence of the TMR ratio and the switching field for magnetisation of MnGa nanolayer *H*_*c*_.

To obtain the insight into the mechanism of the low TMR ratio in the present p-MTJ, the temperature variation of the TMR effect was investigated. Figure 5(a) shows TMR curves for different measurement temperatures. The TMR curves exhibit well-defined anti-parallel states owing to the large difference of the switching field between the CoFeB and MnGa layers. The TMR ratios are 12.8%, 9.7%, and 3.1% at 5 K, 100 K, and 300 K, respectively. The TMR ratio as a function of the measurement temperature is shown in Fig. 5(b). It increases with decreasing temperature, which is consistent with results seen in the MTJs with conventional materials^1^,^2^,^3^,^4^. The temperature dependence of the switching field *H*_*c*_ of the MnGa layer is also shown in Fig. 5(b). The variation of *H*_*c*_ for the MnGa layer in the figure is larger than that observed in thick MnGa films^24^, which could be partially explained by a small reduction of the Curie temperature *T*_*c*_ and/or two-dimensionality of the thin-layer, as a significant increase of *H*_*c*_ is also observed for the top CoFeB layer with PMA in Fig. 5(a). In addition, the rate of increase of the TMR ratio from RT to low temperature is nearly the same as for the thick MnGa-based MTJs^40^, suggesting that *T*_*c*_ for the 3-nm-thick MnGa film is not significantly reduced. The thermal fluctuation of magnetisation of Mn atoms at the MnGa/MgO interface, which reduces the spin-polarisation of current by spin-flip scattering, may not be dominant, either, because the TMR effect shows saturation at low temperature and there are no abnormal increases at low temperature observed in MTJs with a half-metallic Heusler alloy, such as Co_2_MnSi^42^.

As for the temperature dependence of *H*_*c*_, it may be necessary to take into account the magnetism of CoGa and the possible magnetic exchange coupling of the CoGa and MnGa layers at the interface because CoGa shows ferromagnetism at low temperature. The *T*_*c*_ of CoGa depends on composition and chemical ordering, and is typically ~100 K for CoGa with B2 order and the present composition^43^. The temperature dependence of *H*_*c*_ for the MnGa layer shows a monotonic change without any anomalies, which implies a lack of significant magnetic interaction between the MnGa and CoGa layers and that the CoGa buffer layer seems to behave as a non-magnetic buffer layer, even at low temperature.

## Discussion

### Role of strain

One important insight obtained in this study is the role of epitaxial strain for the MnGa layer. For instance, Köhler *et al*. reported that the PMA constant for a 3-nm-thick Mn-Ga grown on a Cr buffer layer decreased below 1 × 10^6^ erg/cm^3^ and discussed the cause of this in terms of the epitaxial strain induced by the lattice mismatch between the Cr buffer and MnGa layer^26^. However, the strained MnGa layer still shows a 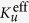 over 5 Merg/cm^3^ in the present study, suggesting that such low epitaxial strain does not dominantly influence the PMA. The intrinsic bulk PMA *K*_*u*_ for the MnGa layer in this study is evaluated to be 7.1 ± 1.1 × 10^6^ erg/cm^3^, which is only a little bit lower than the *K*_*u*_ of 11.5 × 10^6^ erg/cm^3^ for the 30-nm-thick MnGa grown on a Cr buffer^16^. This dependence of PMA on the small strain is in accordance with the first principles calculation^44^.

**Figure 6:**
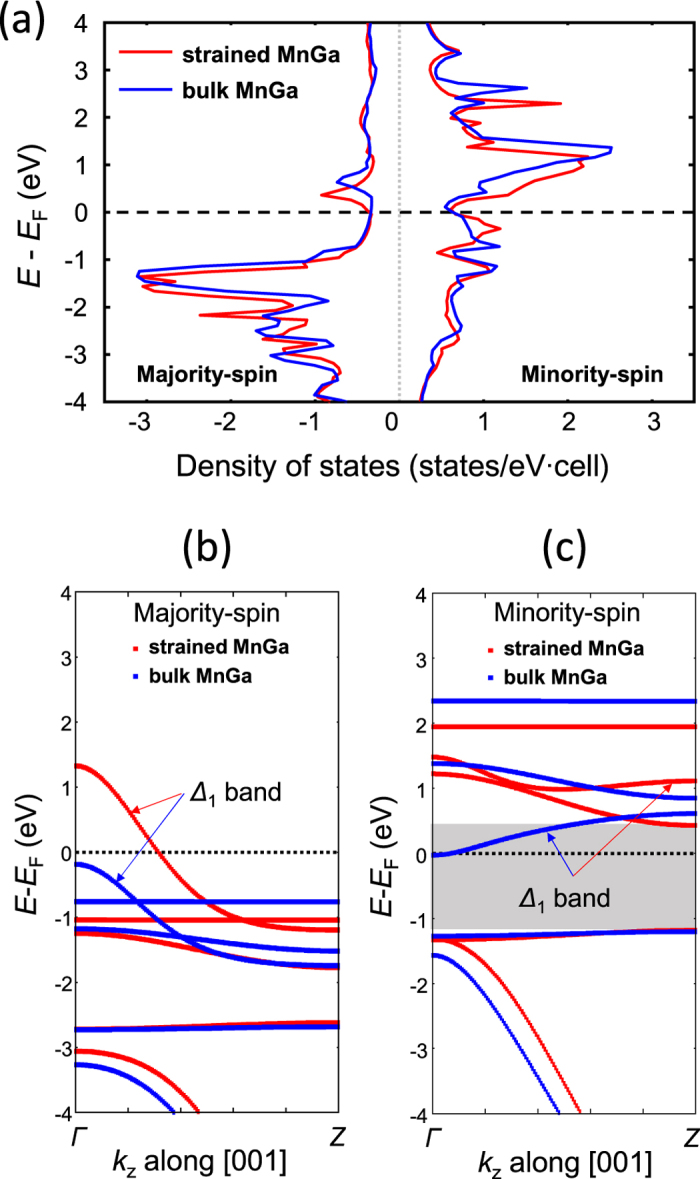
First principles electronic structure calculation of MnGa. The data for MnGa with a bulk and strained tetragonal unit cell are indicated by the blue and red solid curves, respectively. (**a**) The spin-resolved density-of-states, and the band dispersion along the *c*-axis near the Fermi level *E*_*F*_ for the (**b**) majority and (**c**) minority spin states. The lattice parameters used were those shown in Table. 1. The dashed lines represent *E*_*F*_ . The energy bands of Δ_1_ symmetry are denoted by the arrows. The gray area represents the band gap in the minority spin band for the strained MnGa.

On the other hand, this epitaxial strain results in an interesting modification of the band structure for MnGa. The electronic structure calculations for the bulk and strained MnGa are shown in Fig. 6, where the lattice parameters used in the calculation were those shown in Table 1. The small reduction of tetragonal distortion only slightly changes the whole profile of the density of states, as seen in Fig. 6 (a). Figure 6(b,c) display, respectively, the majority and minority spin-resolved band dispersions along the *c*-axis for MnGa, i.e., the Γ − *Z* line in the Brillouin zone. Bulk MnGa has no states and almost negligible state for the majority and minority spin sub-band at *E*_*F*_. On the other hand, the strained MnGa has a fully spin-polarised band with the Δ_1_ symmetry in the majority spin band and a band gap in the minority spin band, similar to the case of Mn_3_Ge^32^,^33^,^34^. This is because the small change of tetragonal lattice shifts the energy levels for both spin states at the Γ point near *E*_*F*_. Such band gap of Δ_1_ band in the minority spin is not seen in the total density-of-state because the states with the other k points near the Fermi energy overlap. In addition, the strained growth of MnGa in the present MTJs reduces the lattice mismatch between the MnGa layer and MgO barrier to 5.4% from 8.5% for the bulk case, which is favourable for the growth of MgO the barrier with fewer misfit dislocations. This band structure and lattice matching should lead to the condition of exhibiting huge TMR ratios in the p-MTJs with strained MnGa layers, being similar to the huge TMR effect predicted and observed in the Co-Fe/MgO system with a lattice mismatch of ~5%^3^,^4^,^30^,^31^. However, the TMR ratio was not increased significantly in the present MTJs and was still comparable to that of the p-MTJ composed of thick MnGa/MgO/CoFeB reported previously^40^.

### Source for reduction for TMR

As mentioned above, the mismatch between the strained MnGa and MgO is similar to that for Fe(Co)/MgO, and the MgO barrier seems to have a well-crystalline structure in the TEM image, as shown in Fig. 2(a). In addition, we also examined the reference MTJs of Cr/Fe(1)/Mg(0.4)/MgO(2)/CoFeB(1), similar stacking structure, except for the bottom Fe layer, in which the TMR ratio of about 35% was evaluated even without annealing (see Supplemental information). Thus, it is unlikely that the imperfection of MgO barrier and the un-annealed CoFeB layer are the dominant origin of significantly small TMR ratio in the present MnGa MTJs.

One origin of reduction of TMR effect could be discussed in terms of the influence of less-chemical ordering and slightly off-stoichiometric composition of the present MnGa nanolayer. The net magnetisation of MnGa is insensitive to the strain found from the first principles calculation in Fig. 6. *M*_*s*_ can be reduced by decreasing the L1_0_ chemical ordering, such as site swapping of Ga at the corner site and Mn centred at body in the unit cell shown in Fig. 1, because Mn occupying at the original Ga site has a magnetic moment antiparallel to that of Mn occupying at the original Mn site^16^. Magnetisation in the present MnGa layer is a little bit lower than 420 emu/cm^3^ for the thick film with an L1_0_ ordering parameter of approximately 0.7 obtained by post-annealing^16^. Thus the chemical ordering of the MnGa layer might be slightly less than this value, likely due to RT growth. Moreover, the slightly off-stoichiometric composition results in the excess Mn atom occupying at the original Ga site, which might change the Δ_1_ band structure.

Other origin of the low TMR ratio may be the effect of ferrimagnetic Mn spin possibly existing at interface. Recently it was proposed that the TMR effect may significantly depend on the elements terminating the interface of Mn_3_Z (Z = Ga, Ge)/MgO. Miura *et al*. predicted the spin-polarisation of tunneling current relevant to the Δ_1_ band is strongly reduced in the case of Mn-Ga atomic plane termination in the p-MTJ of Mn_3_Ga/MgO/Mn_3_Ga, and that it shows negligible TMR effect^33^. They also theoretically suggested that it is not the case in the Mn_3_Ge/MgO/Mn_3_Ge p-MTJs which show termination independent huge TMR ratio because of the existence of the fully spin-polarised band along the *c*-axis^33^. It should be noted that the theoretical calculation implied that the strained MnGa/MgO/MnGa p-MTJs also exhibit termination independent huge TMR ratio, similar to Mn_3_Ge case. On the other hand, Jeong *et al*. quite recently reported the experimental TMR ratio of approximately −30% at RT in the Mn_3_Ge/MgO/CoFeB p-MTJs and they explained that its small/negative TMR ratio results from the sign of spin-polarisation depending on the direction of the magnetic moment of the Mn terminating the interface of Mn_3_Ge/MgO, i.e. the positive or negative spin-polarisation for the Mn-Mn or Mn-Ge atomic plane terminations, respectively, both of which probably exist in the real MTJs due to atomic level roughness and then cancel the net spin-polarisation^34^. These discussions may be relevant to the low TMR ratio in the present study, because the Mn atoms possibly located in the Ga atomic plane terminating the interface may tend to reduce the net spin-polarisation. This interface issue remains a fundamental topic to be addressed by clarifying the physics of the Mn-based alloy/MgO hetero-interface^34^,^45^.

The above discussions indicate that interface engineering to form pure Mn or Ga atomic plane termination as well as precise control of composition and L1_0_ chemical ordering can lead to huge TMR ratios in p-MTJs with a strained MnGa nanolayer and it remains as a future technological challenge.

In conclusion, we have demonstrated p-MTJs with a MnGa nanolayer, exhibiting high PMA and low saturation magnetisation, which has been never seen in p-MTJs with other materials. This p-MTJ fabrication was achieved with the aide of a unique buffer layer material CoGa and RT growth methods. The atomic level TEM and high-energy X-ray measurements revealed the atomically defined interfaces and well-ordered crystalline structure of the MnGa nanolayer with an epitaxial strain. The first principles calculations showed that the strained MnGa has a fully spin-polarised band structure along the *c*-axis. These results indicated that the strained MnGa is very beneficial for advanced STT-applications, such as STT-MRAM and ultra high-frequency STT-oscillator/diode devices. The TMR ratio for this p-MTJ was still low but comparable to that of the p-MTJs with much thicker MnGa electrodes. The origins of reduction of TMR ratio were duscussed and the enhancement of TMR ratio is further subject.

## Method

### Sample preparation

The MTJ stacking structure of MgO substrate/Cr(40)/CoGa(30)/MnGa(3)/MgO(2)/CoFeB(1)/Ta(3)/Ru(5) was prepared by an ultra-high vacuum sputtering system under a base pressure of below 1 × 10^−7^ Pa. The film composition of CoGa and MnGa were evaluated by inductively coupled plasma mass spectroscopy (ICP-MS). The reference films of Cr(40)/CoGa(30)/MnGa(3)/MgO(5) and Cr(40)/CoGa(30)/MgO(2) were also prepared for the magnetisation and XAS measurements. All the layers were deposited at RT and the heating treatments were performed only for the MgO substrate and buffer layers. The 0.4-nm-thick ultrathin Mg layer was deposited prior to the MgO deposition to prevent oxidation of the bottom layer. The details of the deposition process have been described elsewhere^29^. The microfabrication of MTJs were carried out by standard ultra-violet photo-lithography and Ar ion mill etching. The junction area of the MTJs ranged from 10 × 10 to 100 × 100 *μ*m^2^. Ti/Au and SiO_2_ were used for the counter electrode and interlayer insulating material, respectively. The MTJs were not intentionally annealed.

### Sample characterisation

The HRTEM and HAADF-STEM for atomic structure characterisation were performed with an acceleration voltage of 200 kV (JEM-ARM200F, JEOL). The beam spot diameter for the nano-beam electron diffraction was approximately 2 nm.

The magnetic properties were characterised using a polar MOKE system and vibrational sample magnetometer with magnetic field of 20 kOe. Electrical transport properties were investigated with a physical property measurement system (PPMS, Quantum Design) using a four probe method under applied magnetic field up to 90 kOe and in the temperature range of 5–300 K.

The XAS and XMCD analyses were performed at BL-7A and BL-16A in the Photon Factory at the High-Energy Accelerator Research Organization (KEK). The photon helicity was fixed, and a magnetic field of ±12 kOe was applied parallel to the incident polarised soft X-ray beam. The total electron yield mode was adopted, and all measurements were performed at RT with an incident beam energy resolution of *E*/Δ*E* = 2000. The XAS and XMCD measurement geometries were set to normal incidence, so that both the photon helicity and the magnetic field were normal to the surface.

### Electronic structure calculations

First-principles density-functional calculations of the spin-polarised band structures were performed using the Vienna ab-initio simulation package (VASP)^46^. The exchange-correlation functional was taken within the generalised gradient approximation (GGA) and the parametrisation of Perdew-Burke-Ernzerh to the density functional theory (PBE)^47^. The band structure was also examined by the scalar-relativistic full potential linearised augmented plane wave (FLAPW) method with GGA-PBE^48^.

## Additional Information

**How to cite this article**: Suzuki, K. Z. *et al*. Perpendicular magnetic tunnel junction with a strained Mn-based nanolayer. *Sci. Rep.* **6**, 30249; doi: 10.1038/srep30249 (2016).

## Supplementary Material

Supplementary Information

## Figures and Tables

**Table 1:** The summary of the parameters for crystal structures.

Materials	*a* (nm)	*c* (nm)	*c*/*a*	*v* (nm^3^)	reference
CoGa	0.286	—	—	—	bulk^35^
	0.287	0.284	—	—	this work
MnGa	0.275	0.364	1.32	0.0275	bulk^36^
	0.286	0.340	1.19	0.0278	this work
MgO	0.422	—	—	—	bulk^37^
	0.426	0.420	—	—	this work

*a* and *c* are the in-plane and out-of-plane lattice parameters, respectively, for CoGa, MnGa, and MgO layers in the p-MTJ evaluated from the electron diffraction patterns. *c*/*a* is the axial-ratio and *v* is the unit cell volume. The unit cells are taken as shown in Fig. 1. The bulk values are also included for comparison.
