# Supplementary material for: Perpendicular magnetic tunnel junction with a strained Mn-based nanolayer
Source: Sci Rep. 2016 Jul 26;6:30249. doi: 10.1038/srep30249 (PMC4960582; doi:10.1038/srep30249)
Supplement: Supplementary Information [file srep30249-s1.pdf]

Supplementary information

Perpendicular magnetic tunnel junction with a strained Mn-based nanolayer

K. Z. Suzuki,[1, a)] R. Ranjbar,[1] J. Okabayashi,[2] Y. Miura,[3] A. Sugihara,[1] H. Tsuchiura,[4] and S. Mizukami[1]

1)*WPI Advanced Institute for Materials Research, Tohoku University, Sendai 980-8577,Japan*

2)*Research Center for Spectrochemistry, University of Tokyo, Tokyo 113-0033, Japan*

3)*Department of Electronics, Kyoto Institute of Technology, Kyoto 606-8585, Japan*

4)*Department of Applied Physics, Tohoku University, Sendai 980-8579, Japan*

Corresponding Author [a)]: Kazuya Suzuki, kazuya.suzuki.d8@tohoku.ac.jp

1. Tunnel magnetoresistance (TMR) effect for the reference magnetic tunnel junction (MTJs)

The MTJs of the conventional ferromagnetic electrode were fabricated for the reference using the sputtering system same as that for fabrication of the present MnGa-MTJs. The stacking structure of MTJ is (100) MgO substrate/Cr(40 nm)/Fe(1 nm)/Mg(0.4 nm)/MgO(2 nm)/CoFeB(1 nm)/cap layer, in which all the layers were deposited at room temperature and the stacking was not annealed, except for the substrate and Cr buffer. Then the MTJ was patterned into the junction, similar to the MnGa-MTJ. Figure S1 shows the out-of-plane TMR curve of this MTJ without annealing, which has been measured with applying field perpendicular to the film plane using the four-probe method at room temperature. The TMR value is about 15%, where the top CoFeB and bottom Fe layers show perpendicular and in-plane magnetization, respectively. The value of TMR for AP-P state was estimated to be more than 30% by the relation of the angular dependence of TMR effect. The TMR ratio of the reference MTJs is much larger than that of MnGa-MTJs in the manuscript.

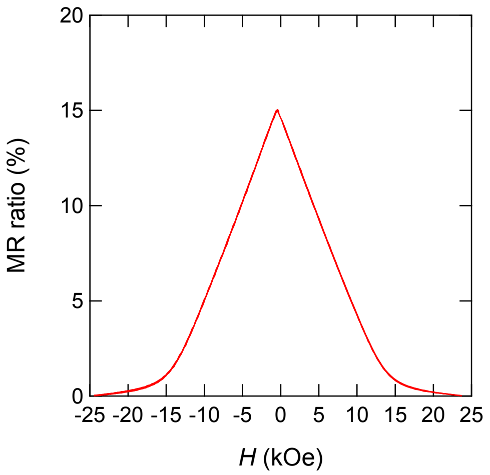

Figure S1 Out-of-plane TMR curve of the reference MTJs.
